# Microstructural abnormalities in deep and superficial white matter in youths with mild traumatic brain injury

**DOI:** 10.1016/j.nicl.2019.102102

**Published:** 2019-11-19

**Authors:** Sonja Stojanovski, Arash Nazeri, Christian Lepage, Stephanie Ameis, Aristotle N. Voineskos, Anne L. Wheeler

**Affiliations:** a Neuroscience and Mental Health Program, Hospital for Sick Children, Toronto, Ontario, Canada; b Department of Physiology, University of Toronto, Toronto, Ontario, Canada; c Mallinckrodt Institute of Radiology, Washington University School of Medicine, St. Louis, MO, USA; d Toronto Rehabilitation Institute, Toronto, Ontario, Canada; e Department of Psychiatry, University of Toronto, Toronto, Ontario, Canada; f The Margaret and Wallace McCain Centre for Child, Youth and Family Mental Health, Centre for Addiction and Mental Health, Toronto, Ontario, Canada; g Campbell Family Mental Health Institute, Centre for Addiction and Mental Health, Toronto, Ontario; h Institute of Medical Science, University of Toronto, Toronto, Ontario, Canada

**Keywords:** Mild traumatic brain injury, Superficial white matter, Diffusion tensor imaging, Processing speed

## Abstract

**Background:**

Diffusion Tensor Imaging (DTI) studies of traumatic brain injury (TBI) have focused on alterations in microstructural features of deep white matter fibers (DWM), though post-mortem studies have demonstrated that injured axons are often observed at the gray-white matter interface where superficial white matter fibers (SWM) mediate local connectivity.

**Objectives:**

To examine microstructural alterations in SWM and DWM in youths with a history of mild TBI and examine the relationship between white matter alterations and attention.

**Methods:**

Using DTIDWM fractional anisotropy (FA) and SWM FA in youths with mild TBI (TBI, n=63) were compared to typically developing and psychopathology matched control groups (n=63 each). Following tract-based spatial statistics, SWM FA was assessed by applying a probabilistic tractography derived SWM mask, and DWM FA was captured with a white matter fiber tract mask. Voxel-wise z-score calculations were used to derive a count of voxels with abnormally high and low FA for each participant. Analyses examined DWM and SWM FA differences between TBI and control groups, the relationship between attention and DWM and SWM FA and the relative susceptibility of SWM compared to DWM FA to alterations associated with mild TBI.

**Results:**

Case-based comparisons revealed more voxels with low FA and fewer voxels with high FA in SWM in youths with mild TBI compared to both control groups. Equivalent comparisons in DWM revealed a similar pattern of results, however, no group differences for low FA in DWM were found between mild TBI and the control group with matched psychopathology. Slower processing speed on the attention task was correlated with the number of voxels with low FA in SWM in youths with mild TBI.

**Conclusions:**

Within a sample of youths with a history of mild TBI, this study identified abnormalities in SWM microstructure associated with processing speed. The majority of DTI studies of TBI have focused on long-range DWM fiber tracts, often overlooking the SWM fiber type.

## Highlights

• Deep and superficial white matter structure was altered in youths with mild TBI.
• Slower processing speed was associated with superficial white matter structure in TBI.
• Some abnormalities were more pronounced in superficial compared to deep white matter.

## 1 Introduction

The rapid acceleration and deceleration of the head that occurs during mild traumatic brain injury (TBI) produce torsion, tension, and compression forces within the brain, which can lead to traumatic axonal injury and progressive white matter pathology (Armstrong et al., 2016). Diffusion tensor imaging (DTI) studies have identified that fractional anisotropy (FA) is often decreased in white matter in participants with history of TBI (Hulkower et al., 2013; Roberts et al., 2014), and animal studies have demonstrated a direct correspondence between traumatic axonal injury and decreases in white matter FA (Mac Donald et al., 2007)*.* More than half of all TBIs occur in youths younger than 24 years of age (Rutland-Brown et al., 2006) when white matter development is in progress. Injury to the still-developing brain, can impact ongoing neurodevelopmental processes (Beauchamp and Anderson, 2013) and lead to cognitive impairment (Emery et al., 2016). Though the majority of youths fully recover after a mild TBI, impaired cognitive functioning, including problems with attention, has been reported up to two years post-mild TBI in 29% of a youth sample (Lambregts et al., 2018). Despite a sizable proportion of youths who experience persistent cognitive compromise following mild TBI, there are no existing tools that can predict persistent impairment. Thus, it is imperative to develop methods to better characterize white matter injury and identify those who are at risk of incomplete recovery.

The majority of DTI studies in people with TBI have focused on alterations in microstructural features of deep white matter fibers (DWM) (Hulkower et al., 2013), the long-range fiber bundles that connect the different lobes and hemispheres of the brain, as well as carry signal from the peripheral nervous system into the brain. However, post-mortem studies have demonstrated that injured axons are often observed in areas of changing tissue density, such as the subcortical gray-white matter interface, where shorter superficial white matter fibers (SWM) mediate local connectivity (Catani et al., 2012; Grady et al., 1993; Peerless and Rewcastle, 1967; Povlishock, 1993). SWM fibers makeup 57% of cortical white matter volume (Schuz and Braintenberg, 2002) and mediate local connectivity in the form of U fibers or longer intralobar fibers (Catani et al., 2012; Yeterian et al., 2012). These axons may be particularly vulnerable to injury in youths due to the relatively late myelination of SWM, which continues into the third decade of life (Oyefiade et al., 2018; Reeves et al., 2005). We are aware of no other study that has specifically examined SWM microstructure *in vivo* in youths exposed to TBI.

The pattern of brain damage that results from mild TBI is highly variable and influenced by several factors: the mechanism of injury, injury biomechanics (Ji et al., 2015), and characteristics of the individual, including previous injury, age, genetic factors, and neck strength (Bigler et al., 2013). However, common analytical approaches, i.e., anatomical and voxel-based analyses (such as tract-based spatial statistics (TBSS)) make assumptions about common spatial locations of FA changes in the brain. Case-based methods that have been applied in TBI studies in in adults (Ling et al., 2012; Lipton et al., 2012), veterans (Jorge et al., 2012; Lepage et al., 2018; Miller et al., 2016), and one study in youths (Mayer et al., 2012) capture diffuse and spatially nonoverlapping white matter abnormalities. The composition of the comparison group is another important consideration in the design of neuroimaging studies of TBI. A recent systematic review reported that *de novo* inattentiveness and hyperactivity, elevated mood symptoms, and disruptive behaviors, are common persistent forms of psychopathology experienced following mild TBI in youths (Emery et al., 2016). Additionally, rates of pre-injury psychopathology in youths with mild TBI are more prevalent than in uninjured controls and this psychopathology may predispose youths to persistent post-injury impairment (Max et al., 1997). Various psychopathologies have been associated with widespread abnormalities in FA (Thomason and Thompson, 2011), including SWM-FA abnormalities (Nazeri et al., 2015).

In this study, we hypothesized that SWM fibers are vulnerable to traumatic injury in youths due to their late myelination and path through changes in tissue density (Grady et al., 1993; Peerless and Rewcastle, 1967; Povlishock, 1993). We compare DTI indices in youths with mild TBI to both a typically developing control group (without TBI or other psychopathology), as well as a second control group matched for symptoms of psychopathology, to account for the potential contribution of psychopathology to white matter alterations. We applied case-based voxel-wise methods designed to detect and quantify diffuse heterogeneous patterns of white matter abnormalities. Our objectives were to 1) test for differences in SWM and DWM microstructure across groups; 2) assess for associations between the extent of SWM and DWM microstructural alterations with accuracy and response time in an attention task in youths with mild TBI; and 3) examine the relative susceptibility of SWM and DWM to microstructural alterations found following mild TBI.

## 2 Methods

### 2.1 Sample

The three study groups were composed of participants from the Philadelphia Neurodevelopmental Cohort, a publically available population-based sample of youths aged 8 to 22 years (Calkins et al., 2015). The institutional review boards of both the University of Pennsylvania and the Children's Hospital of Philadelphia approved all study procedures. Signed informed consent was provided by all participants 18 and over and for participants under age 18 assent and parental consent were obtained. Within this cohort, 1445 cohort participants underwent multimodal neuroimaging (Satterthwaite et al., 2014) and completed a computerized neurocognitive battery (Gur et al., 2014) that included the Penn continuous performance attention task (Gur et al., 2012), as well as a structured interview, the GOASSESS, which screened for psychopathology and included a comprehensive medical history (Calkins et al., 2015).

Participants were selected as candidates for analyses if they had complete data to determine the history of TBI. Mild TBI was defined as a reported history of TBI with no skull fracture or neurosurgical intervention that was associated with one or more of the following: loss of consciousness for 30 minutes or less, amnesia for 24 hours or less, or new-onset, post-TBI headaches. Of 89 youths where history was consistent with the definition for mild TBI, 1 was excluded due to excessive motion (defined by visual inspection and identification of artifacts consistent with motion), and 2 were excluded due to poor image quality. Of 1,222 youths without TBI 22 were excluded due to excessive motion and 25 were excluded due to poor image quality. Participants were then excluded if they had any serious medical conditions from which 63 youths with mild TBI and 1079 youths without a history of TBI remained. A typically developing control pool (N=381) was extracted from the total pool of youths with no history of TBI and no evidence of significant psychopathology (symptoms, treatment, or hospitalization). Group matching was done in an unbiased, blinded fashion with the R package MatchIt through the application of nearest neighbor matching (at a one-to-one ratio) based on covariate distributions (Ho et al., 2007). Using individual matching to the mild TBI group on mean age, sex proportion, and mean level of parental education, a final typically developing control group of 63 youths was selected. A psychopathology matched control group was derived from the remainder of the pool of youths. This group comprised TBI-free youth not extracted to form the matched typically developing control group and had sufficient psychopathology data (N=1010). This group was additionally matched to the TBI on the number of internalizing and externalizing symptoms (reported on the GOASSESS), as well as the number of participants meeting criteria for psychosis spectrum Gur et al., 2014 to create the final psychopathology matched control group of 63 youths.

### 2.2 Diffusion tensor imaging

**Fig. 1:**
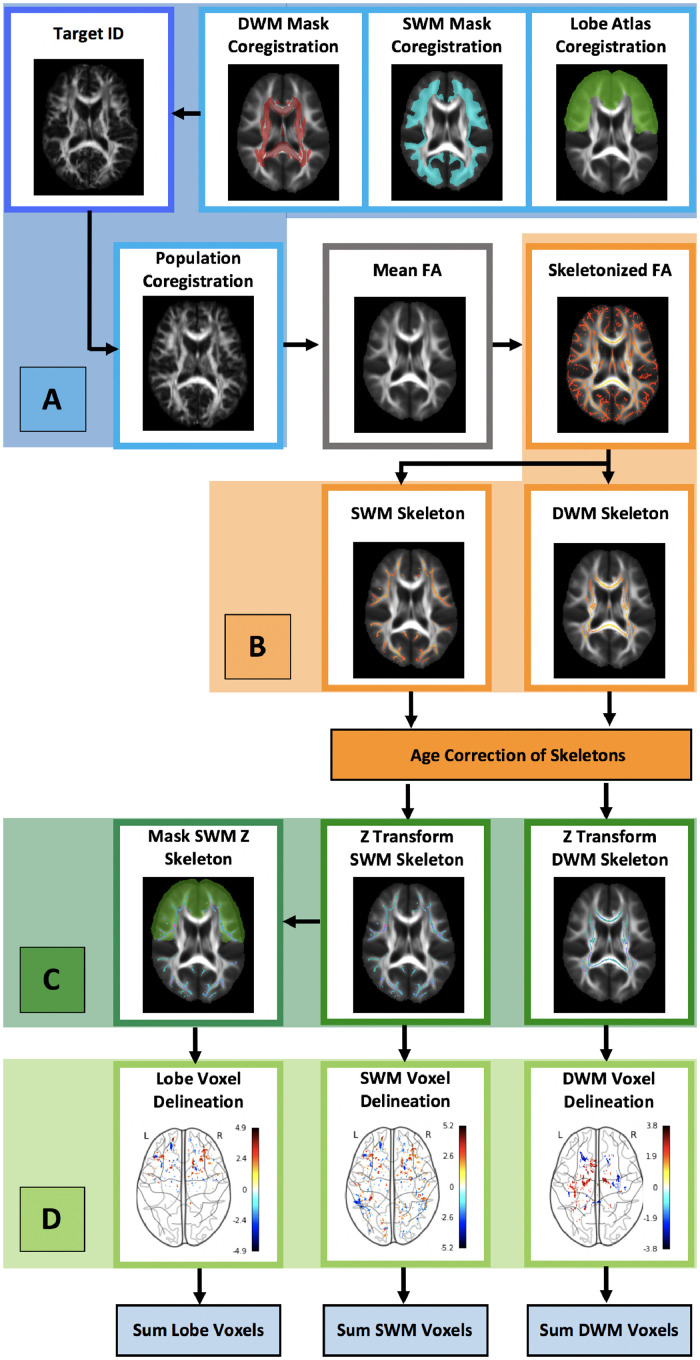
Summary of methods for detecting abnormal FA in deep and superficial white matter. (A) COREGISTRATION: Native space FA images were nonlinearly registered to each other to identify the most representative participant of the population (target ID). The target FA image was brought to MNI152 space, and the population, DWM and SWM masks, and MNI structural atlas (lobe atlas, frontal lobe used as an example) [43] were co-registered to this target. The co-registered population was then used to create a population mean-FA image. (B) CREATION OF SWM AND DWM SKELETONS: The mean-FA image was skeletonized to generate a white matter representation of the centers of all tracts common to all subjects (skeletonized FA) and thresholded at FA > 0.15 for SWM or FA > 0.2 for DWM. Skeletons were masked with DWM and SWM masks to create the SWM and DWM FA skeletons which were then corrected for age. (C) Z TRANSFORM FA SKELETONS: Typically developing controls FA skeletons for DWM and SWM were Z transformed with a leave one-out-approach, while all other participants skeletons were transformed using the typically developing control population mean and standard deviation for each voxel. Lobes within the SWM were defined masking the Z transformed age-corrected SWM skeleton with the registered and binarized lobar atlas (frontal lobe used as an example). (D) THRESHOLD AND COUNT VOXELS: Voxels with Z > 2 or Z <-2, part of a cluster of at least 2 contiguous voxels were counted within the SWM, DWM, and each of 5 SWM lobes to derive the number of abnormally high and low voxels in each substrate.

All participants were scanned on the same 3T Siemens TIM Trio scanner. The diffusion-weighted MRI acquisitions were obtained using a twice-refocused spin-echo single-shot EPI sequence with 64 diffusion-weighted directions with b=1000 s/mm^2^, and 7 scans with b=0s/mm^2^ in 2mm slices. Acquisition parameters are described in detail by Satterthwaite et al. (Satterthwaite et al., 2014). Processing and analysis steps were performed to compute voxelwise FA in a DWM and SWM skeleton and assess the number of voxels with abnormally low or high FA in each participant. These steps are illustrated in Fig. 1 and described below.

#### 2.2.1 Preprocessing

Diffusion-weighted scans were concatenated and corrected for motion, and eddy current distortions with FSLs eddy correct (version 6.0.0). Following this correction, FSL brain extraction was performed (Smith, 2002), and FA for all voxels was calculated by fitting the diffusion tensor model in each voxel using FSL's dtifit function (FMRIB's Diffusion Toolbox, implemented in FSL).

#### 2.2.2 Augmented TBSS

The TBSS pipeline, described in detail by Smith et. al was augmented to extract SWM-FA and DWM-FA (Smith et al., 2006). SWM-FA extraction is described in detail by Nazeri et al. (2015), as well as briefly below. Following the generation of the population mean FA image, FA was thresholded to remove non-white matter at 0.2 for DWM, and 0.15 for SWM to include finer superficial structures that would otherwise disappear at greater threshold values (Bach et al., 2014). Though a lower threshold may lead to some false-positives in the TBSS skeleton (Bach et al., 2014) constraining the analysis using a probabilistic tractography-derived SWM-mask (described below), mitigates this risk. To account for residual misalignments and facilitate group-wise comparison, each individual's FA image was skeletonized as has been previously described (Smith et al., 2006), resulting in a SWM-FA or DWM-FA skeleton for each participant.

#### 2.2.3 White matter subtype segmentation

DWM was defined as white matter included in the JHU atlas and was extracted by binarizing the JHU atlas in MNI152 space then masking the DWM skeleton. SWM is both adjacent to the cortex and is not included in any of the deep white matter regions of the John Hopkins University (JHU) labels (Wakana et al., 2007) and was captured by the application of a SWM mask described in detail and generated by Nazeri et al. (2015). Briefly, a probabilistic tractography derived SWM mask was generated in a separate population of 141 typically developing participants without a history of head trauma, neurological or mental disorders, or evidence of substance use. Cortical reconstruction was performed on the structural T1-weighted (Fischl, 2012) scans of the participants of this separate population to extract the grey matter - white matter boundary of both hemispheres, which was nonlinearly registered to the diffusion space of each individual in this population. Probabilistic tractography was conducted via seeding along the grey matter white matter boundary, and tractography results were normalized for each individual in this population, based on the total number of successfully traced streamlines, transformed to standard space, and averaged across subjects. The resulting SWM mask was brought into MNI152 space. The 4D SWM-FA skeleton from the population explored in this study was masked to isolate SWM.

#### 2.2.4 Subject-specific FA analysis

Prior to generating the z score maps, the FA skeleton was corrected for age, and FA values were adjusted at the voxel-level using a fitted general linear model when the model including the effect of age was significant (Lepage et al., 2018; Lipton et al., 2012). For each participant, a profile of white matter abnormality was generated by computing a voxel-wise z-score map over the DWM and SWM FA age-corrected skeleton. Skeletons were normalized in typically developing controls using a leave-one-out approach to reduce the risk of false positives introduced by reference groups with non-independence (Lepage et al., 2018; Watts et al., 2014). Normalized skeletons were generated for all groups based on the mean and standard deviation of the typically developing control group. Skeletons were then thresholded at Z >2 or Z < 2 to generate individualized maps of high or low FA respectively (Ling et al., 2012; Mayer et al., 2012). Next, voxels that belonged to clusters with a minimum of 2 contiguous voxels were summed to generate the number of voxels in DWM or SWM with low and high FA (Jenkinson et al., 2012).

#### 2.2.5 SWM lobe definition

Lobes within the SWM were defined by affine registration of the MNI structural atlas (Mazziotta et al., 2001) to the target participant's FA image (selected for TBSS) (Smith et al., 2006), followed by masking the z-score transformed age-corrected SWM skeleton (2.2.4) with the registered and binarized lobar atlas.

### 2.3 Attention response time and accuracy

The continuous performance task of the Penn computerized neurocognitive battery assessment (Gur et al., 2012) was used to assess attention. Response time was calculated as the median response time in the assessment of continuous performance for all correct responses, such that higher values indicated poorer performance, and accuracy was calculated as the number of correct trials (Weinberger et al., 2016).

### 2.4 Statistics

All statistical analyses were conducted using R software (R version, 3.3.2).

#### 2.4.1 FA abnormalities in SWM and DWM in mild TBI

Negative binomial regression modelling was used to examine the effect of group on the number of voxels with high and low FA in DWM and SWM. The assumptions for this model were assessed and were met (Hilbe, 2014). Evaluation of goodness of fit of each model through the generation of QQ plots confirmed model selection. This analysis included sex, standardized score from the wide range assessment test (WRAT) (Wilkinson and Robertson, 2006), and the highest level of parental education as covariates. The magnitude of the difference between the mean values of voxels with abnormal FA for each substrate is reported in youths with a history of TBI compared to each control group (Δ). Analyses were repeated at a range of Z score thresholds, incremented by 0.1 five times above and below the threshold used for this study to ensure the stability of the results. Analyses were also repeated within 5 bilateral lobes of the SWM (frontal, insular, temporal, occipital, and parietal), to explore regional group differences. Regional analyses were Bonferroni corrected to account for the 5 lobe comparisons such that p<0.01 was considered significant.

#### 2.4.2 Association between FA abnormalities in SWM and DWM and attention

Associations between the number of voxels in DWM or SWM with high or low FA and response time and accuracy on the attention task were examined with general linear regression. This analysis was performed in youths with mild TBI and included sex, WRAT, and the highest level of parental education as covariates. Regional analyses were performed within the 5 bilateral lobes of SWM, and results were Bonferroni corrected to account for comparisons within each lobe, such that p<0.01 was considered significant. Due to the Gaussian assumptions of this parametric model, box cox transformations were applied to the dependent variable in all models to improve Gaussianity and model fit (Box and Cox, 1982)

#### 2.4.3 Relative susceptibility of SWM and DWM to FA abnormalities in mild TBI

The number of voxels with high or low FA in DWM and SWM were normalized to the size of the skeleton and compared with the Wilcoxon signed ranked test with continuity correction*.*

## 3 Results

### 3.1 Sample characterization

Injury characteristics of the mild TBI participants are summarized in Table 1. Age, sex, WRAT, highest level of parental education and temporal signal to noise ratio were compared between the mild TBI and both control groups, and none of these variables were significantly different. As expected, the mild TBI group and psychopathology-matched controls reported more symptoms of internalizing and externalizing disorders and had a higher proportion of youths on the psychosis spectrum compared to the group of typically developing controls (Table 2).

**Table 1:** TBI characteristics in the study population sample.

TBI Characteristics			
Number of TBI, Mean (SD)	1.68 (1.25)		
LOC	YES	NO	UNKNOWN
	28	34	0
LOC minutes, Mean (SD)	1.4 (1.2)		
Amnesia	YES	NO	UNKNOWN
	48	14	1
Amnesia minutes, Mean (SD)	37.1 (188.2)		
Headaches post TBI	YES	NO	UNKNOWN
	28	35	0

Mean values of continuous variables are reported with standard deviations (SD) in brackets. LOC: loss of consciousness.

**Table 2:** Participant characteristics.

	TBI	PMC	TD	P value ANOVA
Age in years, mean (SD)	16.1 (2.9)	16.3 (2.7)	16.1 (2.9)	0.87
Sex, n	29 F, 34 M	31 F, 32 M	34 F, 29 M	0.67
WRAT score, mean (SD)	103.8 (15.8)	102.5 (14.7)	105.7 (13.1)	0.48
Parental education in years, mean (SD)	15.8 (2.8)	15.5 (2.6)	15.8 (2.9)	0.81
TSNR, mean (SD)	7.2 (0.6)	7.1 (0.6)	7.2 (0.6)	0.74
Internalizing disorder symptoms, mean (SD)	13.2 (11.2)	13.9 (10.8)	5.2 (5.1)	2.3e-7
Externalizing disorder symptoms, mean (SD)	9.1 (7.7)	8.4 (7.3)	4.4 (5.2)	2.3e-4
Psychosis spectrum inclusion, n	51 No, 12 Yes	52 No, 11 Yes	63 No	1.4e-3

Mean values of continuous variables are reported with standard deviations (SD) in brackets. P values reflect differences between specified mild TBI group, psychopathology matched controls, and typically developing controls calculated with an ANOVA for continuous variables and Pearson's Chi-Squared test for categorical variables. M: male, F: female. WRAT: IQ was measured using the standardized Wide Range Achievement Test 4 (WRAT-4) scores. Education: highest level of parental education. TSNR: Temporal Signal to Noise Ratio of the diffusion-weighted imaging. Internalizing disorder symptoms: number of endorsed symptoms of agoraphobia, generalized anxiety disorder, major depressive disorder, obsessive-compulsive disorder, panic disorder, phobias, post-traumatic stress disorder, separation anxiety and social anxiety. Externalizing disorder symptoms: number of endorsed symptoms of ADHD, conduct disorder, oppositional defiance disorder and mania. Psychosis Spectrum Inclusion: participants who endorsed sufficient symptoms of positive sub-psychosis, positive psychosis, or negative/ disorganized symptoms. Details on the psychosis spectrum classification can be found in Calkins (Calkins et al., 2014).

### 3.2 Case-based comparison of FA abnormalities in SWM and DWM between mild TBI and controls

#### 3.2.1 Abnormalities found for SWM FA in mild TBI vs. controls

Group comparisons revealed more voxels with abnormally low FA and fewer voxels with abnormally high FA in SWM in youths with mild TBI compared to both typically developing and psychopathology-matched control groups.

**Fig. 2:**
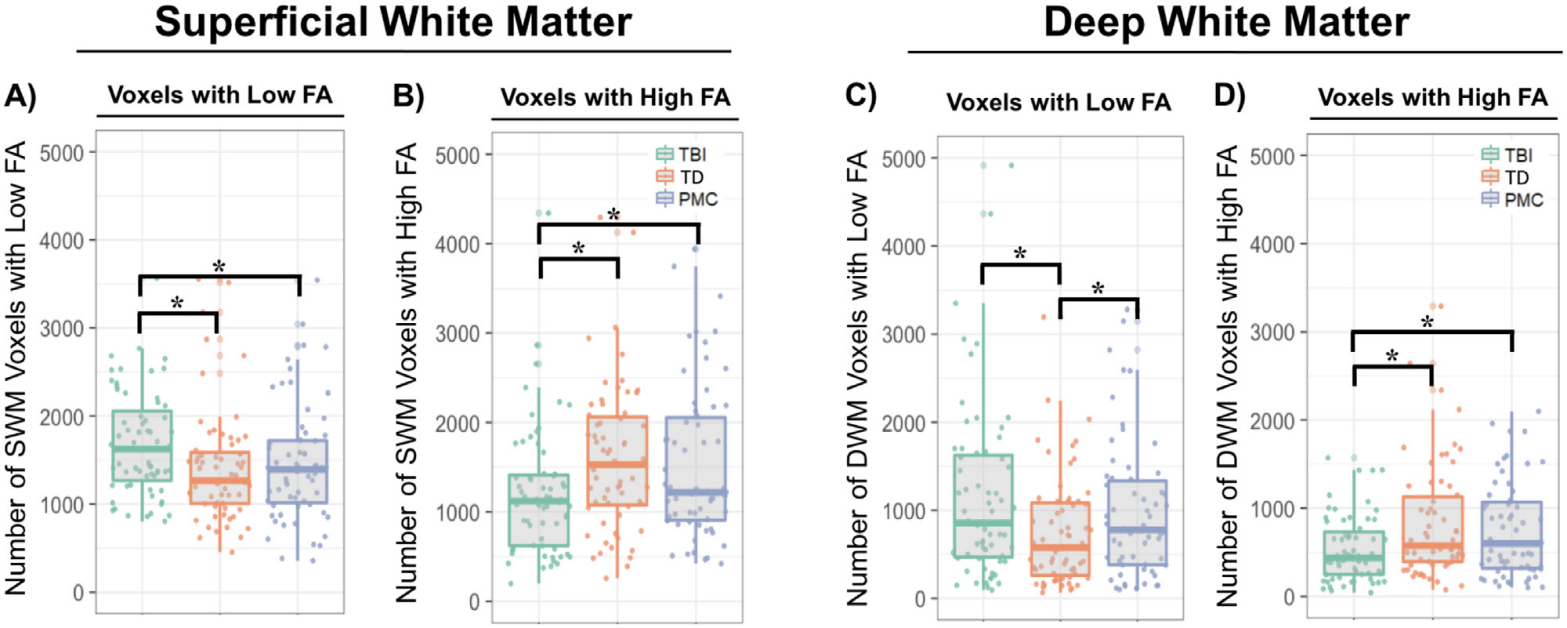
FA abnormalities in youths with mild TBI compared to typically developing (TD) and psychopathology matched controls (PMC). (**A**) There were more voxels with low FA in SWM in youths with mild TBI compared to TD and PMC. (**B**) There were fewer voxels with high FA in SWM in youths with mild TBI compared to TD and PMC. (**C**) There were more voxels with low FA in DWM in youths with mild TBI compared to TD but there were no differences with PMC. (**D**) There were fewer voxels with high FA in DWM in youths with mild TBI compared to TD and PMC. Asterisks indicate significant group differences (P<0.05).

**Fig. 3:**
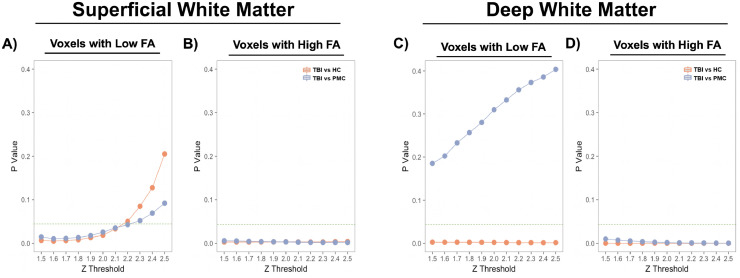
Stability of FA abnormalities in youths with mild TBI compared to typically developing (TD) and psychopathology matched controls (PMC), across Z-score thresholds. Thresholds were incremented by 0.1 five times above and below the threshold used for this study (2.0). (**A**) There were more voxels with low FA in SWM in youths with mild TBI compared to TD and PMC. This result was stable across all Z thresholds below 2.3. (**B**) There were fewer voxels with high FA in SWM in youths with mild TBI compared to TD and PMC across all examined thresholds. (**C**) There were more voxels with low FA in DWM in youths with mild TBI compared to TD, but not PMC. These results were stable across all examined thresholds. (**D**) There were fewer voxels with high FA in DWM in youths with mild TBI compared to TD and PMC. These results were stable across all examined thresholds.

There were more voxels with low FA in the SWM of youths with a history of mild TBI compared to both typically developing controls (z= -2.36, p= 0.019, Δ=17%) and psychopathology matched controls (z= -2.23, p=0.026, Δ=14%) (Fig. 2**A**). This result was stable across all Z thresholds below 2.3 (Fig. 3**A**). Regional analyses of group differences of voxels with abnormally low SWM-FA revealed a greater number of voxels with low FA in the frontal lobe in youths with history of mild TBI compared to typically developing controls (z=-3.63, p=2.89e-4, Δ=28%), but this association did not reach our threshold for significance in psychopathology matched controls (z=-2.28 p=0.02, Δ=17%). There were no significant group differences in the insular, occipital, parietal, or temporal lobes when examining the number of voxels with abnormally low FA (Table 3).

**Table 3:** Group comparisons in SWM lobes (low FA and high FA).

	Low FA				High FA			
	mild TBI-TD		mild TBI-PMC		mild TBI-TD		mild TBI-PMC	
	Z	P	Z	P	Z	P	Z	P
Frontal Lobe	-3.6	2.9e-4	-2.3	0.02	2.6	8.8e-3	2.0	0.04
Insular Lobe	-2.3	0.02	-0.3	0.76	2.4	0.02	2.1	0.03
Occipital Lobe	-0.3	0.77	-1.9	0.05	2.7	6.6e-3	3.1	2.1e-3
Parietal Lobe	-1.3	0.19	-1.6	0.10	2.2	0.03	2.3	0.02
Temporal Lobe	-0.6	0.55	-0.3	0.76	2.6	0.01	2.4	0.01

Group difference Z and P values reflects differences in the number of voxels with low or high FA between the specified groups. Differences were assessed with a negative binomial model that included sex, highest level of parental education, and IQ (WRAT) as covariates. Typically developing controls (TD), psychopathology matched controls (PMC).

There were fewer voxels with abnormally high FA in the SWM of youths with mild TBI compared to typically developing controls (z=2.94, p= 3.31e-3, Δ=28%) and psychopathology-matched controls (z=2.95, p=3.22e-3, Δ=28%) (Fig. 2**C**). These results were stable across all examined Z thresholds (Fig. 3**C**). As with low SWM FA, findings were significant in the frontal lobe. There were fewer voxels with high FA in the frontal lobe in participants with mild TBI compared to typically developing controls (z= 2.62, p= 8.79e-3, Δ=26%) but not psychopathology matched controls (Table 3). Additionally, there were fewer voxels with high FA in the occipital lobe in youths with mild TBI compared to typically developing controls (z=2.72, p= 6.60e-3, Δ=31%) and psychopathology matched controls (z= 3.08, p=2.09e-3, Δ=34%).

#### 3.2.2 Abnormalities found for DWM FA in mild TBI vs. controls

Equivalent group comparisons in DWM revealed a similar pattern of results, except the number of voxels with abnormally low FA in youths with mild TBI was not significantly different from psychopathology matched controls (z= -1.02, p=0.311, Δ=11%). There were more voxels with low FA in the DWM in youths with a history of mild TBI compared to typically developing controls (z= -3.10, p=0.002, Δ=43%; Fig. 2**B**). These results were stable across all examined Z thresholds (Fig. 3**B**).

Comparing the number of DWM voxels with abnormally high FA revealed there were fewer voxels with high FA in youths with mild TBI compared to typically developing controls (z= 4.35, p= 1.34e-05, Δ=54%) and psychopathology matched controls (z= 3.15, p=1.65e-3, Δ=40%) (Fig. 2**D**). These results were also stable across all examined Z thresholds (Fig. 3**D**).

### 3.3 Association between FA abnormalities in SWM and DWM and attention

#### 3.3.1 Response time in the attention task

**Fig. 4:**
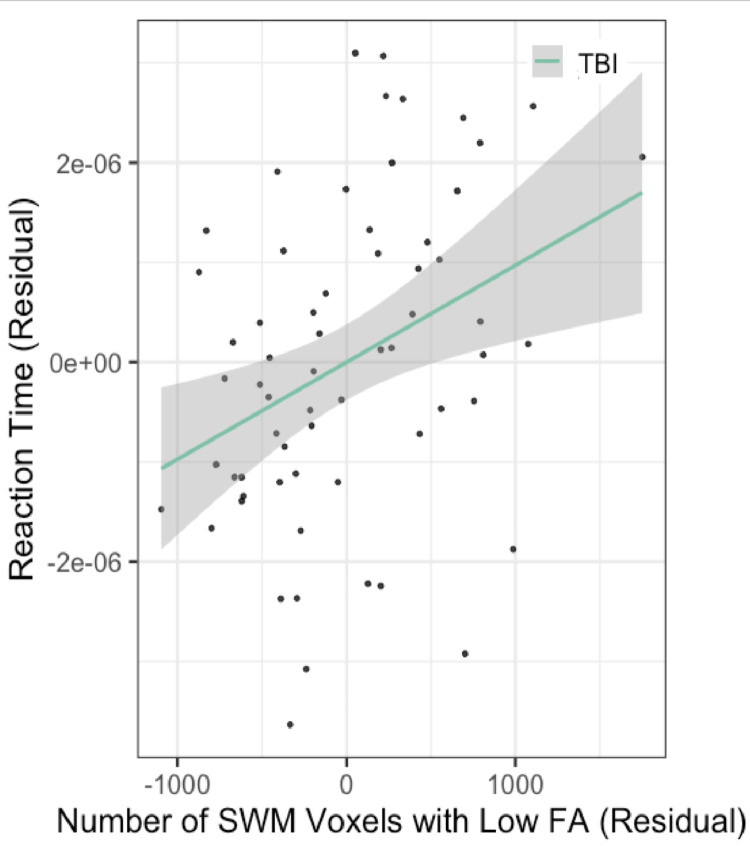
The relationship between the number of SWM voxels with abnormally low FA in youths with mild TBI and response time on an attention task. Residuals after considering model covariates are plotted as relative measures. A significant positive relationship was detected between response time, and the number of SWM voxels with low FA in youths with mild TBI. The regression line is plotted with the shaded area representing 95% confidence intervals for the linear regression.

*Association between FA abnormalities in SWM and response time in the attention task in youths with mild TBI:* The average response time for correct response trials in the attention task was positively correlated with the number of SWM voxels with abnormally low FA in the mild TBI group (t= 2.890, p=5.41e-3; Fig. 4) (Table 4). Regional analyses revealed that within participants with a history of mild TBI, response time was positively correlated with the number of voxels with low FA in the parietal lobes (t=3.172, p=2.42e-3) (Table 5).

**Table 4:** Associations between the number of DWM or SWM voxels with FA abnormalities and processing speed or accuracy.

	SWM				DWM			
	Low FA		High FA		Low FA		High FA	
	T	P	T	P	T	P	T	p
	Processing Speed							
mild TBI	2.9	5.4e-3	-1.11	0.27	2.4	0.02	-1.8	0.07
	Accuracy							
mild TBI	-1.72	0.09	1.18	0.24	-1.50	0.14	1.05	0.30

T and P values from the model that assessed the association between the number of voxels with low or high FA, and processing speed or accuracy on the attention task. Associations were assessed with a linear model that included sex, the highest level of parental education, and IQ (WRAT) as covariates.

**Table 5:** Associations between the number of voxels with FA abnormalities and processing speed or accuracy in SWM Lobes.

	Frontal Lobe		Insular Lobe		Occipital Lobe		Parietal Lobe		Temporal Lobe	
	T	P	T	P	T	P	T	P	T	P
	Processing Speed									
	Low FA									
mild TBI	1.5	0.15	1.7	0.10	2.5	0.01	3.2	2.4e-3	2.5	0.02
	High FA									
mild TBI	-1.1	0.28	-1.1	0.29	-0.37	0.71	-0.69	0.50	-1.1	0.26
	Accuracy									
	Low FA									
mild TBI	-2.2	0.03	-1.6	0.11	-1.4	0.17	-1.2	0.23	-0.49	0.63
	High FA									
mild TBI	1.3	0.21	-0.03	0.97	0.76	0.45	0.50	0.62	1.31	0.19

T and P values from the model that assessed the association between the number of voxels with low or high FA, and processing speed or accuracy on the attention task. Associations were assessed with a linear model that included sex, the highest level of parental education, and IQ (WRAT) as covariates.

Response time on the attention task was not correlated with the number of SWM voxels with abnormally high SWM-FA at a level that reached our threshold for significance. Regional analyses of relationships of high SWM-FA with response time revealed no relationships that survived Bonferroni correction (Table 5).

*Association between FA abnormalities in DWM and response time in the attention task in youths with mild TBI:* The average response time for correct response trials in the attention task was positively correlated with the number of DWM voxels with abnormally low FA in the mild TBI group (t= 2.351, p=0.022). Response time in the attention task was not correlated with the number of DWM voxels with abnormally high FA at a level that reached our threshold for significance. (Table 4).

#### 3.3.2 Accuracy in the attention task

Accuracy in the attention task was not correlated with the number of voxels with abnormally low or high FA in SWM or DWM in youths with mild TBI (Table 4), and this was also the case in post-hoc regional analyses (Table 5).

### 3.4 Relative susceptibility of SWM and DWM to FA abnormalities in mild TBI

**Fig. 5:**
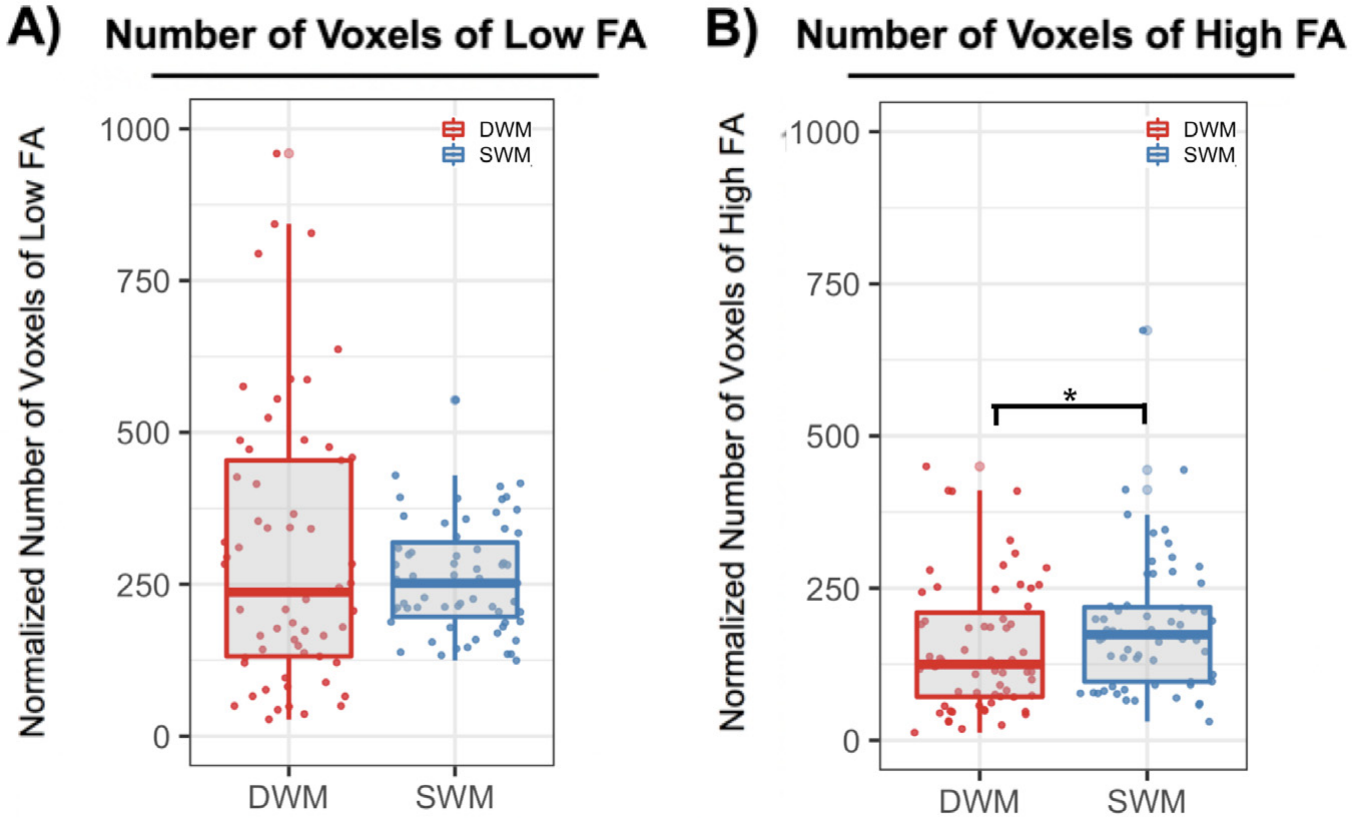
The number of abnormal SWM and DWM voxels in youths with mild TBI. (**A**) Youths with mild TBI did not have different levels of voxels with low FA in DWM compared to SWM. (**B**) Youths with mild TBI had increased levels of voxels with high FA in SWM compared to DWM. The number of abnormal voxels was normalized for SWM and DWM skeleton size.

Youths with mild TBI had a higher proportion of voxels with high SWM FA compared to DWM (v= 504, p=5.66e-4). The proportion of voxels with low FA was not significantly different between SWM and DWM in youths with mild TBI (v=1250, p=0.098) (Fig. 5).

## 4 Discussion

In youths aged 8 to 22 years, we examined (1) the effect of mild TBI on superficial and deep white matter and (2) the association between attention and microstructure in these fiber types in youths with mild TBI. Although several studies have examined the effects of mild TBI on DWM, we know of no other study that has examined the effect of mild TBI on both DWM and SWM, even though mild TBI is thought to have pronounced effects at the white matter cortical interface where SWM fibers are located. Spatially heterogeneous alterations in white matter microstructure were detected in both DWM and SWM in youths with mild TBI. The mild TBI group had more SWM voxels with abnormally low FA and fewer voxels with abnormally high FA, relative to typically developing and psychopathology matched controls. Further, the mild TBI group had more DWM voxels with abnormally low FA than typically developing controls but not psychopathology matched controls, and fewer DWM voxels with abnormally high FA than typically developing and psychopathology matched controls. Processing speed was correlated with SWM and DWM integrity among participants with mild TBI, such that slower response times were correlated with more SWM and DWM voxels of abnormally low FA. The mild TBI group also had more voxels with high SWM FA compared to high DWM FA, which may reflect a compensatory role for SWM following mild TBI in youths.

The separation of DWM from SWM allowed us to investigate diffusion abnormalities within classes of white matter with different function and developmental trajectories (Catani et al., 2012; Oyefiade et al., 2018). Low FA after mild TBI may represent damaged axons, reduced myelination, inflammation, edema, or a combination of these factors (Assaf and Pasternak, 2008). We predicted that SWM fibers would be vulnerable to these types of damage from mild TBI, due to their location at the grey-white matter interface and relatively late myelination in development (Catani et al., 2012; Grady et al., 1993; Oyefiade et al., 2018; Peerless and Rewcastle, 1967; Povlishock, 1993; Reeves et al., 2005). Previous post-mortem studies have identified axonal damage to SWM following mild TBI in youth athletes (McKee et al., 2014) and in individuals with repetitive mild TBI that preceded chronic traumatic encephalopathy (McKee et al., 2015). Accordingly, our *in vivo* findings of increased SWM low FA voxel count in the mild TBI group and its association with processing speed suggests these fibers may play a significant role in mild TBI-related damage and outcome in youths. Short-range SWM fibers mediate local connectivity within lobes that have differing functionality, susceptibility to initial coup-contrecoup injury (Armstrong et al., 2016; Hulkower et al., 2013), and developmental trajectories (Catani et al., 2012; Oyefiade et al., 2018), which motivated the examination of SWM-FA abnormalities within each lobe. Low SWM-FA was most pronounced in the frontal lobes, consistent with previously reported patterns of decreased FA in mild TBI (Hulkower et al., 2013). SWM integrity has previously shown promise as a potential biomarker of cognition, as SWM-FA in the insula and frontal operculum have been associated with processing speed in typically developing adults (Nazeri et al., 2013). The association between SWM-FA in the parietal lobes, which are associated with attention and higher-level motor planning (Bisley and Goldberg, 2010; Critchleey, 1953), and processing speed in youths with mild TBI, suggests that attention and processing speed deficits may be related to SWM damage in the parietal lobe.

Consistent with previous research (Hulkower et al., 2013), relative to typically developing controls, the mild TBI group had more voxels with low FA in DWM. This suggests a detrimental effect of injury on DWM, even when spatially heterogeneous differences are evaluated. This is consistent with a recent modeling study that described the susceptibility of long tracts found in DWM due to high fiber strain that can occur anywhere along the tract (Ji et al., 2015). However, there were no differences in the number of DWM voxels with low FA detected when the mild TBI group was compared to the psychopathology matched control group. Given that mild TBI is associated with pre- and post-injury psychopathology, such as mood disorders, externalizing disorders, and behavioral problems (Emery et al., 2016; Max et al., 1997), and that psychopathology is associated with widespread abnormalities in FA (Thomason and Thompson, 2011), including decreases in SWM-FA (Nazeri et al., 2015), previous studies that focused on DWM may be confounded by tract abnormalities associated with pre-existing psychopathology. Attempts to disentangle the complex relationships between mild TBI, psychopathology, and cognitive impairment require further consideration. Though previous research supports DWM involvement in attention (Stojanovski et al., 2018) and processing speed (Wilde et al., 2006; Wilde et al., 2010), no relationship with DWM fibers was identified in this study. This may be due to a difference in methodology since we compared spatially heterogeneous group differences rather than individual long-range fibers. Ultimately, we did not detect differences in the proportion of voxels with low FA in DWM and SWM suggesting that white matter, whether located deeply within long-range tracts or superficially at the grey matter cortical interface, is equivalently susceptible to injury. It is of interest to note that the range of the number of voxels with low FA was notably larger in DWM than that of the SWM. We speculate that this may be because DWM fiber tracts are more uniformly organized in orientation than SWM. A recent modelling study demonstrated the importance of white matter fiber orientation in the amount of strain experienced by the fibers during a traumatic injury (Ji et al. 2015). This may lead to heterogeneous mechanisms and locations of injury producing more varied amounts of damage to DWM then in SWM, which contains fibers with diverse orientations.

Potential underlying structural contributions to high FA include increased myelination, axon packing density, and branching (Beaulieu, 2002). Consistent with there being more voxels with low FA in the mild TBI group there were fewer voxels with high FA across SWM and DWM when compared to both control groups. In contrast to the low SWM-FA which was specific to the frontal lobe in mild TBI, high SWM-FA was detected across all of the examined lobes (though not all comparisons survived Bonferroni correction). Negative relationships between high DWM FA voxel count with response time across our sample suggest that an increased number of voxels with high FA may be indicative of a recovery process in mild TBI. We found increased high SWM-FA relative to high DWM-FA in the mild TBI group which may point to a post-injury process that is distinct among the two white matter locations. The nature of this process – whether pathological or compensatory – remains to be clarified and further investigation is required to elucidate the differential vulnerability and recovery trajectories of SWM and DWM.

Several factors should be considered when interpreting the results of this study. Our analytical approach differed from the convention (Ling et al., 2012; Lipton et al., 2012; Mayer et al., 2012) by accounting for the spatial inhomogeneity of mild TBI FA changes. Previously, one study took a similar approach and reported more voxels with high DWM-FA in youths with mild TBI; however this was a small sample of youths in the acute stages of recovery and they were compared to typically developing controls (Mayer et al., 2012). An important caveat to the approach of matching groups based on symptoms of psychopathology is that the impact of psychopathology on FA may be different in youths with and without a history of mild TBI, as we have previously shown (Stojanovski et al., 2018). Additionally, the application of tract-based spatial statistics may not be optimal for two reasons. First, the generated mean FA skeleton has been shown to be less ‘alignment-invariant’ than anticipated (Bach et al., 2014), which is not ideal for the investigation of SWM in particular as it is susceptible to the issue of cortical folding variability, Dickie et al. (2018) due to connecting adjacent gyri. Second, SWM heavily features crossing fibers (Reveley et al., 2015), particularly among sulcal depths (Reveley et al., 2015). This may exacerbate a weakness of TBSS in distinguishing between gyri and sulci, as well as between adjacent, differentially oriented fiber bundles with similar FA values in the creation of a mean FA skeleton (Bach et al., 2014). As these methodological considerations may impact the specificity of voxelwise comparisons we mitigated this potential issue by applying a methodology that does not require spatial homogeneity in group differences. Future studies may consider methodology, such as constrained spherical deconvolution based tractography, which is robust to crossing fibers (Alexander, 2005; Descoteaux et al., 2009; Tournier et al., 2007, 2004).

These results support the investigation of SWM as an indicator of structural abnormality due to mild TBI, particularly when concurrent symptoms of psychopathology are present. The functional relevance of abnormalities in SWM is supported by the association between the extent of low SWM-FA and processing speed on an attention task and increased levels of high SWM FA compared to DWM in youths with mild TBI. An increased focus on alterations to this often-overlooked fiber type after mild TBI may provide better detection of injuries, as well as inform the progression of impairment and recovery after injury.

## Disclosures

SA receives financial support from the Centre for Addiction and Mental Health Foundation via the O'Brien Scholarship Fund, and the Academic Scholars Award from the Department of Psychiatry, University of Toronto.

All other authors report no declarations of interest or potential conflicts of interest.

## Declaration of Competing Interest

The authors declare that they have no known competing financial interests or personal relationships that could have appeared to influence the work reported in this paper.
